# Mental health of the old- and new-generation migrant workers in China: who are at greater risk for psychological distress?

**DOI:** 10.18632/oncotarget.15985

**Published:** 2017-03-07

**Authors:** Bao-Liang Zhong, Sandra S.M. Chan, Tie-Bang Liu, Dong Jin, Chi-Yi Hu, Helen F.K. Chiu

**Affiliations:** ^1^ Department of Psychiatry, The Chinese University of Hong Kong, Hong Kong SAR, PR China; ^2^ Affiliated Wuhan Mental Health Center/The Ninth Clinical School, Tongji Medical College of Huazhong University of Science & Technology, Wuhan, Hubei Province, PR China; ^3^ Shenzhen Key Laboratory for Psychological Healthcare, Shenzhen Institute of Mental Health, Shenzhen Kangning Hospital, Shenzhen Mental Health Center, Shenzhen, Guangdong Province, PR China

**Keywords:** migrant worker, psychological distress, epidemiology, generation

## Abstract

Rural-to-urban migrant workers (MWs) are a large vulnerable population in China and, recently, the new-generation MWs (those born in 1980 or later) have become the majority of this population. Examining difference in the epidemiology of poor mental health between the new- and old-generation (those born before 1980) MWs would facilitate mental health promotion efforts. However, very few related studies are available and they produced conflicting findings. This study investigated intergenerational difference in prevalence and correlates of psychological distress (PD) in MWs. A total of 3031 MWs (691 old- and 2340 new-generation MWs) completed a standardized questionnaire containing socio-demographic, migration-related, and work-related variables and the Chinese 12-item General Health Questionnaire (GHQ-12). A GHQ-12 score of 3 or higher was used to denote PD. PD was more prevalent in the new- than old-generation MWs (36.2% versus 28.2%, *P* < 0.001). The elevated risk of PD in the new- versus old-generation remained significant after controlling for potential confounders (OR=1.51, *P* < 0.001). For the new-generation, correlates for PD included low monthly income, recent two-week physical morbidity, migrating alone, poor Mandarin proficiency and long working hours; while for the old-generation, correlates for PD included low education, recent two-week physical morbidity, and having worked in many cities. The new-generation MWs are at higher risk for PD than the old-generation MWs. Mental health services for addressing the generation-specific needs may be an effective way to prevent or reduce PD of MWs.

## INTRODUCTION

Rural-to-urban migrant workers (MWs) are a large population in contemporary China, which accounts for approximately 20% of the total Chinese population [1]. Owing to their socially and economically disadvantageous status in urban China, it is generally believed that MWs are a particularly vulnerable group at high risk for poor mental health [2].

Recently, a growing number of studies in China have examined the association between rural-to-urban migration and mental health [3–12]. These studies point to a complex relationship in which the nature of the correlation between rural-to-urban migration and mental health varies depending on both the particular characteristics of the MWs and mental health indicators used. For instance, prior epidemiological studies [3, 4, 10, 13] suggest that MWs are a heterogeneous group and factors such as age at first migration, duration of migration work, migration pattern (with a partner or alone) and even area of originating are associated with their mental health problems. Therefore, variations in the characteristics of the MW samples may result in inconclusive findings on the migration-mental health relationship.

Mental health is also a heterogeneous construct and the various measures of mental health seem to be differentially associated with migrant status. For example, a comparative study conducted in rural China found a significantly lower prevalence of depressive symptoms in MWs than non-migrants, but a comparable prevalence of poor psychological quality of life and non-fatal suicidal behaviors between the two groups [4]. More importantly, while our previous meta-analysis has provided quite convincing evidence that the prevalence of psychological symptoms tends to be greater among MWs than the Chinese general population, it also revealed a borderline significant declining trend in psychological symptoms over the years of study [5]. Thus the potential effect of changing characteristics of MWs must also be taken into consideration when exploring the migration-mental health link.

China's massive rural-to-urban migration has lasted for nearly four decades [14]. Since the end of the 20th century, a new generation of MWs has emerged and gradually become the majority of the MW population [15]. The term, new-generation MWs, is officially defined as those who were born from 1980 onwards, hold an agricultural *hukou* (household registration identity), and are currently primarily engaged in non-agricultural urban employment [16]. This new generation, which has represented over 60% of the tens of millions of Chinese MWs now [1, 16], is younger, more educated and more urban (e.g., some have a brief urban living experience during childhood) than the old-generation (those born before 1980s). As a result, the new-generation MWs have more ambitious career expectations (i.e., becoming managers and entrepreneurs) and higher demands for material and spiritual enjoyments, and are more cognizant and demanding of the rights to which they are entitled (i.e., annual leave) and the benefits to which their urban peers have access (i.e., medical insurance) [17]. In addition, unlike the old-generation MWs who intend to eventually return to their rural hometowns, the new-generation MWs are more eager to become a part of the urban society [15].

Due to these significant differences, generational status might be another important source of heterogeneity in the migration-mental health association. Therefore, comparing the mental health between the old- and new-generation MWs and further exploring causes underlying such intergenerational difference would provide insights into the nature of MWs’ mental health, help identify high-risk subgroup, and facilitate subgroup-specific mental health promotion efforts, however, only four previous studies have examined this intergenerational difference in China and they produced mixed results [15, 18, 19]. Two studies compared the mental wellbeing and psychological symptoms between the old- and new-generation MWs and found the new-generation had poorer mental wellbeing and more obsessive-compulsive, depressive, anxiety, and hostile symptoms than the old-generation [18, 19]. Yang et al. examined the depressive and anxiety symptoms of the two generations but found no significant intergenerational difference in the severity of depression and anxiety [20]. Another study compared the subjective wellbeing between the two generations and demonstrated that, compared to the old-generation, the new-generation MWs had a higher sense of happiness and were generally more optimistic [15].

Despite these differences, it is true that both generations are experiencing various forms of difficulties during the process of rural-to-urban migration, including financial hardship, institutional exclusion due to their rural *hukou*, hazardous working conditions, discrimination and social isolation [6, 14, 21]. Consequently, the two generation MWs tend to be emotionally stressed by these exposures and experience more psychological distress, which is defined as a state of emotional suffering characterized by symptoms of depression and anxiety [22]. Given these conflicting findings on the relative risk of poor mental health in the old- versus new-generation MWs, the present study investigated the intergenerational difference in prevalence and correlates of psychological distress in Chinese MWs.

## RESULTS

A total of 3140 MWs were invited to participate in the study and 3031 (96.5%) completed. According to the definition of generational status, this sample included 691 (22.8%) old-generation and 2340 (77.2%) new-generation MWs.

Detailed socio-demographic, migration-related and work-related characteristics of the old-and new-generation MWs are shown in Table 1. Compared to the old-generation, the new-generation MWs were significantly more likely to have higher educational attainment, be never-married, migrate before adulthood, originate from Eastern China, have shorter duration of migration work, migrate alone, and have good Mandarin proficiency.

**Table 1:** Socio-demographic, migration-related and work-related characteristics of the old- and new-generation migrant workers

Characteristics		Old generation (n=691)	New generation (n=2340)	χ2	P
Gender	Male	203 (29.4)	488 (32.4)	2.178	0.140
	Female	757 (70.6)	1583 (67.6)		
Education	≥Senior high school	131 (19.0)	995 (42.5)	301.6	<0.001
	Junior high school	422 (61.1)	1272 (54.4)		
	≤Elementary school	138 (20.0)	73 (3.1)		
Marital status	Married	656 (94.9)	939 (40.1)	671.9	<0.001
	Never married	22 (3.2)	1384 (59.1)		
	Others*	13 (1.9)	17 (0.7)		
Average monthly income	≥600 US$	196 (28.4)	637 (27.2)	0.349	0.840
	301-599 US$	433 (62.7)	1490 (63.7)		
	≤300 US$	62 (9.0)	213 (9.1)		
Recent two-week physical morbidity	No	526 (76.1)	1828 (78.1)	1.228	0.268
	Yes	165 (23.9)	512 (21.9)		
Age at first migration	≥18 years	574 (83.1)	1460 (62.4)	103.3	<0.001
	<18 years	117 (16.9)	880 (37.6)		
Geographic region of origin	Eastern China	126 (18.2)	620 (26.5)	30.72	<0.001
	Central China	342 (49.5)	1169 (50.0)		
	Western China	223 (32.3)	551 (23.5)		
Duration of migration work	>10 years	378 (54.7)	205 (8.8)	724.8	<0.001
	≤10 years	313 (45.3)	2135 (91.2)		
No. of cities have worked in	1-2	347 (50.2)	1149 (49.1)	2.735	0.225
	3-4	267 (38.6)	970 (41.5)		
	≥5	77 (11.1)	221 (9.4)		
Migration pattern	With all family members	106 (15.3)	171 (7.3)	56.96	<0.001
	With some family members	376 (54.4)	1192 (50.9)		
	Alone	209 (30.2)	977 (41.8)		
Self-rated Mandarin proficiency	Good	120 (17.4)	527 (22.5)	9.71	0.008
	Moderate	487 (70.5)	1579 (67.5)		
	Poor	84 (12.2)	234 (10.0)		
No. of jobs have engaged in	1-4	572 (82.8)	1973 (84.3)	0.937	0.333
	≥5	119 (17.2)	367 (15.7)		
Average working hours per day	≤8	137 (19.8)	536 (22.9)	2.929	0.087
	>8	554 (80.2)	1804 (77.1)		

*“Others” marital status included remarried, separated, cohabitating, divorced and widowed.

The prevalence of psychological distress (the 12-item General Health Questionnaire [GHQ-12]≥3) was significantly higher in the new- than the old-generation MWs (36.2% versus 28.2%, χ^2^=15.2, *P* < 0.001). After controlling for potential confounders (Table 2), relative to the old-generation, the new-generation MWs still had an increased risk for psychological distress (Odds ratio [OR]: 1.51, 95% confidence interval [CI]: 1.18, 1.93, *P* < 0.001).

**Table 2:** Multiple logistic regression on the unique association of generational status with psychological distress

Variable	Risk level	Reference level	Coefficient	Standard error	χ2	P	OR (95%CI)
Generation	New	Old	0.409	0.126	10.597	0.001	1.51 (1.18, 1.93)
Gender	Male	Female	0.097	0.090	1.158	0.282	1.10 (0.93, 1.32)
Education	≤Elementary school	≥Senior high school	0.208	0.178	1.373	0.241	1.23 (0.87, 1.74)
	Junior high school	≥Senior high school	-0.184	0.095	3.801	0.051	0.83 (0.69, 1.00)
Marital status	Others*	Married	0.163	0.389	0.175	0.675	1.18 (0.55, 2.52)
	Never married	Married	0.003	0.099	0.001	0.973	1.00 (0.83, 1.22)
Average monthly income	≤ 300 US$	≥ 600 US$	0.362	0.159	5.146	0.023	1.44 (1.05, 1.96)
	301-599 US$	≥ 600 US$	0.215	0.097	4.86	0.027	1.24 (1.02, 1.50)
Recent two-week morbidity	Yes	No	0.564	0.091	38.047	<0.001	1.76 (1.47, 2.10)
Age at first migration	<18 years	≥18 years	0.117	0.091	1.652	0.199	1.13 (0.94, 1.35)
Geographic region of origin	Western China	Eastern China	0.245	0.112	4.794	0.029	1.28 (1.03, 1.59)
	Central China	Eastern China	0.054	0.099	0.299	0.584	1.06 (0.87, 1.28)
Length of migration work	≤10 years	>10 years	0.028	0.122	0.054	0.816	1.03 (0.81, 1.31)
No. of cities have worked in	≥5	1-2	0.186	0.147	1.605	0.205	1.20 (0.90, 1.60)
	3-4	1-2	0.108	0.084	1.626	0.202	1.11 (0.94, 1.31)
Migration pattern	Alone	With all family members	0.198	0.156	1.616	0.204	1.22 (0.90, 1.65)
	With some family members	With all family members	0.095	0.148	0.407	0.523	1.10 (0.82, 1.47)
Self-rated Mandarin proficiency	Poor	Good	0.475	0.145	10.696	0.001	1.61 (1.21, 2.14)
	Moderate	Good	-0.016	0.099	0.025	0.874	0.98 (0.81, 1.20)
No. of jobs have engaged in	≥5	1-4	0.095	0.113	0.711	0.399	1.10 (0.88, 1.37)
Average working hours per day	>8	≤8	0.362	0.102	12.528	<0.001	1.44 (1.18, 1.76)

*“Others” marital status included remarried, separated, cohabitating, divorced and widowed.

In multiple logistic regressions (Table 3), correlates for psychological distress of the old-generation MWs included an educational attainment of elementary school or lower, recent two-week physical morbidity, and having worked in more than two cities; while correlates for psychological distress of the new-generation MWs included an average monthly income of 300 US$ or lower, recent two-week physical morbidity, migrating alone, poor Mandarin proficiency and working more than eight hours per day.

**Table 3:** Multiple logistic regression on correlates of psychological distress among the old-and new-generation migrant workers

Variable	Risk level	Reference level	Coefficient	Standard error	χ2	P	OR (95%CI)
Old-generation							
Education	≤Elementary school	≥Senior high school	0.512	0.238	4.630	0.031	1.67 (1.06,2.28 )
Recent two-week physical morbidity	Yes	No	0.472	0.195	5.869	0.015	1.60 (1.09,2.35 )
No. of cities have worked in	≥5	1-2	0.623	0.274	5.148	0.023	1.86 (1.09,3.19 )
	3-4	1-2	0.481	0.185	6.774	0.009	1.62 (1.13,2.32 )
New-generation							
Average monthly income	≤300 US$	≥600 US$	0.236	0.107	4.899	0.027	1.27 (1.03,1.56 )
Recent two-week physical morbidity	Yes	No	0.638	0.103	38.074	<0.001	1.89 (1.55,2.32 )
Migration pattern	Alone	With all family members	0.362	0.184	3.851	0.048	1.44 (1.01,2.07 )
Self-rated Mandarin proficiency	Poor	Good	0.713	0.163	19.189	<0.001	2.04 (1.48,2.81 )
Average working hours per day	>8	≤8	0.377	0.112	11.318	0.001	1.46 (1.17,1.82 )

## DISCUSSION

Intergenerational difference in health is an interesting topic in migrant epidemiology. For example, two studies conducted in Israel and the United States reported similarly high rates of common mental disorders in the first- and second-generation international migrants [23, 24], but a study conducted in Germany found a significantly lower rates of depression, anxiety, and suicidal ideation in the second- than the first-generation immigrants [25]. These intergenerational differences could be related to the second-generation's adaptation to the specific social environment of a host country, therefore intergenerational differential is a potentially useful measure of the effectiveness of a country's social integration policy.

To the best of our knowledge, this is the first large-scale comparative study in China to examine the intergenerational disparities in mental health of Chinese MWs. We found a significantly higher prevalence of psychological distress in the new- than the old-generation MWs and the increased risk in the new-generation remained significant after controlling for a variety of socio-demographic, migration-related, and work-related variables. Our regression analyses further showed the intergenerational differentials in correlates of psychological distress, for instance, migration pattern and Mandarin proficiency were related to psychological distress of the new-generation MWs, but not their old-generation counterparts.

The disadvantage of mental health in the new-generation versus the old-generation MWs found in our study is in keeping with the recent concern about the poor mental health in the new-generation MWs, mainly because the MWs who attempted suicide and died by suicide in the 2010 Foxconn Suicide Cluster belonged to the post-80s generation [26]. Therefore, the present study provided empirical evidence for the poor mental health of the new-generation MWs in terms of psychological distress, which is in line with findings from two previous Chinese MW studies [18, 19].

The elevated risk of psychological distress of the new-generation in comparison to the old-generation MWs may be explained by the age effect. For example, an international migrant study reported that the increased risk of depression and anxiety in Mexican migrants was seen among those aged under 35 years only [27]. However, results of our comparative analysis show that significant generational differences exist, in terms of education, marital status, age at first migration, originating area, length of migration work, migration pattern, and Mandarin proficiency, supporting the notion that the two generations of MWs are distinct entities, rather than two different age-groups [28]. In fact, unlike their predecessors, a great majority of the new-generation MWs lack farming experience and have lower attachment to the rural land [28]. As a result, age effect can not fully explain the intergenerational difference in the prevalence of psychological distress. As confirmed by the results of analyses on the correlates of psychological distress in this study, compared to the old-generation, more correlates were identified to be associated with psychological distress of the new-generation MWs, potentially resulting in the higher risk of psychological distress in the new-generation MWs. Further, intergenerational differences in correlates of psychological distress may indicate some generation-specific risk factors have contributed to the generation gap in the epidemiology of psychological distress.

Although both generations of MWs are paid low wages, relative to the old-generation, the new-generation MWs tend to have much higher expenditures on leisure activities and cultural life [16]. The small income makes it difficult to meet their high demands for entertainment [5]. In this case, low income should have more profound negative effect on the mental health of the new-generation MWs. Therefore, we found, despite similar income levels between the two generations, low income was significantly associated with psychological distress of the new-generation MWs.

MWs are generally engaged in labor-intensive work, which has low educational requirement [2]. Because young MWs often lack working skills, their income levels and job performance are largely determined by the number of working hours and goods produced. On the contrary, the old-generation MWs have accumulated a lot of working experience due to their long duration of migration work (as shown in Table 1). Well-educated individuals of the old-generation MWs may have acquired better working skills and knowledge, therefore working pressure of the old-generation MWs may be not as high as that of the new-generation MWs. Consequently, in our study, low education was related to psychological distress of the old-generation MWs only and long working hours was associated with psychological distress of the new-generation MWs only.

Previous studies have shown that the new-generation MWs have inadequate social support and weak social network, which significantly contribute to their poorer mental health compared to the old-generation MWs [19, 29]. Similarly, our study found migrating alone was significantly correlated with psychological distress of the new-generation MWs. The low level of social support in MWs who migrate alone could explain the association between migrating alone and psychological distress of the new-generation MWs. Nevertheless, the phenomenon, migrating alone was not a correlate of psychological distress of the old-generation MWs, does not mean that migrating alone has no negative effect on the mental health of the old-generation MWs. Perhaps, family support from spouses may offset the negative effect of migrating alone in the old-generation MWs, since our data showed that a significantly higher proportion (96%) of the old-generation MWs had been married.

In international migrant research, proficiency in the host country language is often used as a proxy measure of acculturation and migrants who can not speak fluent host country language have poorer social relationships and higher acculturative stress and, in turn, more depressive symptoms [30–32]. This study also has a similar finding on the relationship between Mandarin proficiency and psychological distress of the new-generation MWs. The finding, no significant link between self-rated Mandarin proficiency and psychological distress in the old-generation MWs, may indicate that language problem is no longer an issue for the old-generation, because they may have adapted the new environment well due to longer duration of migration work.

Finally, we found that the old-generation MWs who had worked in many cities had higher risk of psychological distress, but this finding was not replicated in the new-generation MWs. For the old-generation MWs, having worked in many cities may mean an unstable job status, which in turn can result in higher level of stress due to frequent changes in working environment [3]. However, for the new-generation MWs, having worked in many cities might not be an issue, even means more job opportunities and an increase in the knowledge about what one sees and hears, therefore no significant relationship between the number of cities and psychological distress was observed in the new-generation MWs.

Findings from the present study have implications for social and healthcare policies of mental health promotion in Chinese MWs. Given the trend that the new-generation MWs are replacing the old-generation MWs, efforts to reduce psychological distress among MWs may be useful to target the new-generation group, addressing their specific needs such as providing social support, reducing working load, and increasing monthly wages. Two recent studies showed a very low rate of mental health services utilization of MWs with mental health problems [3, 33]. Therefore, it may be necessary to improve MWs’ mental health literacy and enhance their access to professional mental health care services. In addition, despite lower prevalence of psychological distress in the old- than the new-generation MWs, compared to prior population-based studies using GHQ-12 [34–36], the prevalence of psychological distress (28.2%) was still higher in the old-generation MWs than the Chinese generation population (12.8-21.7%). Thus the mental health problems of the old-generation MWs also deserve special attention.

This study has several limitations. First, this survey was conducted in manufacturing factories of an affluent large city in southern China. MWs of other industries, i.e., service industry, and other cities were not included in this study. Therefore, the generalizability of our findings may be limited. Second, the mental health advantage in the old-generation versus the new-generation MWs may result from a healthy worker effect [37], a healthy migrant effect [38], or both. Due to the poor living and working conditions of MWs, rural-to-urban migration in China could also be regarded as a process of healthy selection: only those who are robust enough to withstand the rigors of migration work, can stay and work long in the cities. A sample of MWs who have returned to rural hometowns is needed to test this possibility. Third, although intergenerational differences in correlates of psychological distress could explain the prevalence disparity in psychological distress, generational status remained significant in our adjustment analysis, which included the above correlates of both generations as confounders. Therefore, there should be some unknown factors that are related to the intergenerational difference. More studies are warranted to explore these unknown factors.

In summary, the new-generation MWs have significantly higher prevalence of psychological distress than the old-generation MWs. Part of this mental health disadvantage can be ascribed to intergenerational differences in correlates of psychological distress. Amongst the two generations of MWs, a range of socio-demographic, migration-related and work-related factors are associated with their psychological distress, indicating the complex and multifaceted nature of mental health problems of MWs. Mental health services for addressing the generation-specific needs may be an effective way to prevent or reduce psychological distress of MWs.

## MATERIALS AND METHODS

### Subjects and sampling

Between August 2012 and January 2013, a large-scale cross-sectional survey was conducted in Shenzhen, the largest migrant city in China's southeast coast. This study adopted multistage sampling approach to obtain a representative sample of factory MWs. Details of sampling, subject recruitment, data collection, and quality control measures have been described in two published articles [3, 39]. In brief, 10 factories of various industry categories were purposively selected to represent factories of Shenzhen in stage one of the sampling. Among the 397 production units of the 10 factories, a total of 41 production units (2-6 units per factory) were randomly selected in stage two of the sampling. Finally, all eligible subjects of these identified units were invited to join this survey. Eligible subjects were workers registered as rural residents, aged 16 years or older, working in manufacturing factories, and living in Shenzhen at the time of the survey.

The study protocol was approved by the Survey and Behavioral Ethics Committee of the Chinese University of Hong Kong. This study was performed in accordance with the ethical standards laid down in the 1964 Declaration of Helsinki and its later amendments. All participants provided written informed consent prior to their inclusion in the study.

### Procedures

Prior to the main study, a pilot study was performed to test our survey questionnaires and procedures. In the formal survey, the survey team leader gave all MWs a brief introduction on the study and instructions for filling out the questionnaires, then these subjects were required to independently and anonymously complete the questionnaires. Trained investigators were assigned to read out questions in the questionnaires for participants who were illiterate or had difficulties in reading. These trained investigators checked the questionnaires for illogical responses or missing values before the collection of questionnaires.

### Measures

The primary mental health outcome, psychological distress, was measured with the Chinese GHQ-12. The GHQ is often considered as the Gold standard for the assessment of psychological distress and has been used in a wide variety of clinical and non-clinical settings [40]. The GHQ-12 items use a dichotomous scale (“0=not at all”, “0=same as usual”, “1=rather more than usual”, and “1=much more than usual”) to rate the extent to which participants have experienced each symptoms during the last few weeks. The Chinese GHQ-12 is reliable and valid in Chinese population and a score of 3 or above is used to denote the presence of psychological distress [34].

Correlates of psychological distress examined in the questionnaire fell into three categories: socio-demographic, migration-related, and work-related variables.

(a) Socio-demographic variables included age, gender, education, marital status, average monthly income, and physical health. Physical health was assessed with the two-week physical morbidity [41]. This health indicator is directly adapted from China's Multi-wave National Health Services Surveys, which asks whether the respondent has experienced any physical health problems, including infectious diseases and chronic non-communicable diseases during the past two weeks.

(b) Migration-related data were age at first migration, geographic region of origin (eastern, central and western China) [42], duration of migration work, total number of cities that MWs had worked in, migration pattern (alone, with some family members, and with all family members), and self-rated standard Mandarin (the official language in China) proficiency (good, moderate, and poor).

(c) Work-related factors included total number of jobs that MWs had engaged in and average working hours per day.

### Statistical analysis

Prevalence of psychological distress by generation was calculated. Chi-square test was used to compare rates of psychological distress and socio-demographic, migration-related and work-related characteristics between the old- and new-generation MWs. The association between psychological distress and generation was investigated in multiple logistic regression model that entered generational status and all socio-demographic, migration-related, and work-related characteristics at once to adjust for potential confounders. To determine the intergenerational difference in correlates of psychological distress, additional multivariable logistic regressions with a backward stepwise entry of all socio-demographic, migration-related, and work-related factors were conducted for the old- and new-generation MW samples, respectively. ORs and 95%CIs were calculated for each variable. The statistical significance level was set at *p* < 0.05 (two-sided). SPSS software version 17.0 package was used for analyses.
